# In This Issue

**DOI:** 10.1002/cfg.177

**Published:** 2002-06


**Comparative and Functional Genomics**
*Comp Funct Genom* 2002; **3**: 219–220.
Published online 9 May 2002 in Wiley InterScience (www.interscience.wiley.com). **DOI:** 10.1002/cfg.177

### In this issue

### Upstream – news in genomics

We report on the genomes that have recently been completed, including the fission yeast (*Schizosaccharomyces pombe*) and rice (*Oryza sativa*). Functional genomics papers include two large-scale studies of *Saccharomyces cerevisiae* protein complexes. Also of interest was a proteomic analysis of blood samples that correctly discriminated patients with ovarian cancer from healthy individuals and those with non-malignant disease.

### Stress response in pine and the Expresso microarray experiment management system

Lenwood Heath *et al.* report on a microarray study of the response to drought stress in loblolly pine, using their Expresso microarray experiment management system. The system supports the whole process, incorporating experiment design, data acquisition, image processing, statistical analysis and data mining. Whilst Expresso can support any method of data analysis, this particular study explores the use of inductive logic programming (ILP).

### Overlapping antisense transcription in the human genome

Maria Fahey and colleagues present a computational search for overlapping antisense transcripts (OATs) of human genes. They compared each complete processed mRNA sequence from RefSeq against the rest of RefSeq and then removed those hits that were solely due to repeat sequences. They identified 56 pairs of overlapping transcripts, and estimate that there could be in the region of 1000 OATs in the human genome.

### Mimotopes and proteome analyses using human genomic and cDNA epitope phage display

Brian Mullaney and colleagues evaluated genomic and cDNA phage display strategies to identify genes in the 5q31 Interleukin gene cluster and to enrich cell surface receptor tyrosine kinase genes from a breast cancer cDNA library. They also discuss the problems caused by bacterial sequences that can act as 'mimotopes' (mimetic sequences of true epitopes).

### Meeting Review: O' Reilly Bioinformatics Technology Conference

Damian Counsell reviews selected presentations from the O' Reilly Bioinformatics Technology Conference. This diverse meeting included talks on topics ranging from BLAST programming and other sequence analysis tools, to data visualization, to computing strategies for proteomics.

### Meeting Review: 2nd Bioinformatics Industrialization Workshop: Bioinformatics and Medicine

Roslin Russell reports on the joint IBM Deep Computing Institute, IUPAB Task Force on Bioinformatics, and EBI conference on Bioinformatics and Medicine. The scope of the meeting was broad, with presentations on subjects such as virtual human biology, computational genomics and proteomics, and E-medicine.

### Meeting Review: Epigenetics in Development and Disease

We present a report from the Keystone symposium on Epigenetics in Development and Disease (which shared some sessions with the symposium on RNA interference, Cosuppression and Related Phenomena) by Andy Mungall. The presentations discussed include those from the sessions on the role of DNA methylation in human disease, the role of DNA methylation in development, and epigenetic mechanisms in tumorigenesis.

### Website Review: How to get the best from fission yeast genome data

Valerie Wood and Jürg Bähler present an overview of the resources currently available (or under

Copyright © 2002 John Wiley & Sons, Ltd.

development) for working with the *S. pombe* genome data. This includes information and advice on making database submissions, accessing *S. pombe* data, choosing gene names, updating and correcting database submissions, and functional genomics resources.

Copyright © 2002 John Wiley & Sons, Ltd.

